# Recurrent Intracranial Atherosclerotic Disease (ICAD)-Large-Vessel Occlusion (LVO) in Acute Lymphoblastic Leukemia Treated by Rescue Angioplasty: A Case Report

**DOI:** 10.7759/cureus.100174

**Published:** 2025-12-27

**Authors:** Ryunosuke Yoshihara, Tomoaki Ishizuka, Masahiro Okuma, Tatsushi Hatayama, Hirohiko Nakamura

**Affiliations:** 1 Department of Neurosurgery, Nakamura Memorial South Hospital, Sapporo, JPN; 2 Department of Neurosurgery, Nakamura Memorial Hospital, Sapporo, JPN

**Keywords:** acute lymphoblastic leukemia (all), intracranial atherosclerotic disease, mechanical thrombectomy (mt), percutaneous transluminal angioplasty (pta), secondary stroke prevention

## Abstract

Malignancy-associated stroke results from diverse cancer-related coagulopathies, and therapeutic options remain limited beyond management of the underlying malignancy. In particular, a large-vessel occlusion (LVO) that occurs despite multiple antithrombotic agents is difficult to attribute to routine atherothrombotic mechanisms and warrants consideration of occult malignancy.

An 80-year-old man presented with left hemiparesis and dysarthria. Magnetic resonance imaging revealed an acute infarction in the right middle cerebral artery territory, and magnetic resonance angiography demonstrated right M1 occlusion. Mechanical thrombectomy using a combined technique achieved reperfusion. Whole-body computed tomography revealed splenomegaly, and rapidly progressive pancytopenia subsequently prompted bone marrow examination, confirming Philadelphia chromosome-positive acute lymphoblastic leukemia (ALL). Hematuria with worsening anemia required discontinuation of antiplatelet therapy. Subsequently, recurrent occlusion of the M1 segment of the right middle cerebral artery occurred. Repeat thrombectomy achieved reperfusion, and a platelet-rich white thrombus was retrieved. Intracranial percutaneous transluminal angioplasty (PTA) was performed for residual stenosis, resulting in satisfactory luminal expansion.

This case underscores the importance of suspecting malignancy-associated coagulopathy, rather than antithrombotic resistance, when LVO occurs despite multiple antithrombotic agents, and highlights the need for prompt systemic evaluation. Although this is a single case and additional cases need to accumulate, this case suggests that even when conventional antithrombotic therapy is not feasible due to malignancy-related bleeding risk, revascularization strategies may offer potential benefit in cancer-associated arterial thrombosis.

## Introduction

Major subtypes of ischemic stroke include cardioembolic stroke due to atrial fibrillation and large-artery atherosclerotic stroke associated with atherosclerosis [1]. For secondary prevention of stroke, anticoagulants are generally used for the former, whereas antiplatelet agents are used for the latter. In addition to medical therapy, revascularization is a therapeutic option for atherosclerotic stroke. However, the optimal treatment for symptomatic intracranial atherosclerotic disease (ICAD), particularly in cases with recurrent events during the acute phase, remains highly controversial [2-4].

In contrast, in malignancy-associated stroke, some reports suggest potential benefits of antithrombotic therapies such as warfarin, direct oral anticoagulants, unfractionated or low-molecular-weight heparin, and antiplatelet agents, including aspirin. However, the optimal treatment strategy remains controversial, and no established therapy exists apart from management of the underlying malignancy [5]. Furthermore, some malignancies cause a bleeding tendency, making antithrombotic therapy difficult to continue for secondary stroke prevention [6]. This issue is especially significant in hematologic malignancies, where bleeding risk seems to be further increased due to coagulation abnormalities and thrombocytopenia.

In the present case, the patient presented with a large-vessel occlusion (LVO) despite receiving both antiplatelet and oral anticoagulant therapy and was subsequently diagnosed with acute lymphoblastic leukemia (ALL) after whole-body computed tomography (CT) revealed a lesion suspicious for malignancy. Due to the bleeding tendency, continuation of antithrombotic therapy became difficult, and the patient subsequently experienced recurrent ischemic stroke. Intracranial percutaneous transluminal angioplasty (PTA) performed during the acute phase was effective in this case of symptomatic ICAD.

## Case presentation

**Figure 1:**
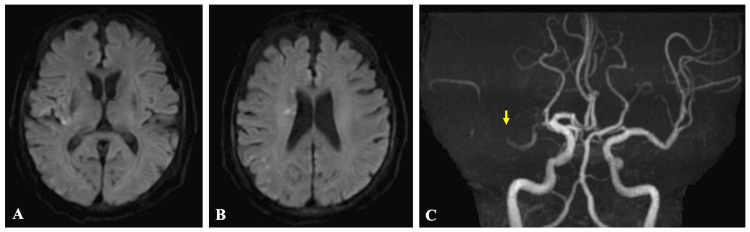
(A and B) Brain magnetic resonance imaging and (C) angiography at admission Axial diffusion-weighted images show hyperintense lesions involving a portion of the right middle cerebral artery territory (A and B). Magnetic resonance angiography demonstrates occlusion of the M1 segment of the middle cerebral artery (C, arrow)

An 80-year-old man was brought to the emergency department with sudden-onset left hemiparesis and dysarthria, with an onset-to-door time of 106 minutes. He had no pre-existing functional disability, with a prestroke modified Rankin scale (mRS) score of 0. On arrival, he was E3V4M6 on the Glasgow Coma Scale, and his National Institutes of Health Stroke Scale (NIHSS) was 12. Diffusion-weighted magnetic resonance imaging (MRI) revealed an acute infarct in the right middle cerebral artery (MCA) territory, and the Alberta Stroke Program Early CT Score (ASPECTS) was 8 (Figures 1A-1B). It also demonstrated an insular cortex negative sign, which is considered characteristic of ICAD-LVO. Magnetic resonance angiography (MRA) showed an occlusion of the proximal M1 segment of the right MCA (Figure 1C).

He had a history of atrial fibrillation and was taking edoxaban 60 mg and prasugrel 3.75 mg for ischemic heart disease. Intravenous alteplase was considered inappropriate because his prothrombin time-international normalized ratio was 4.52. Mechanical thrombectomy (MT) was selected, with a door-to-puncture time of 99 minutes.

**Figure 2:**
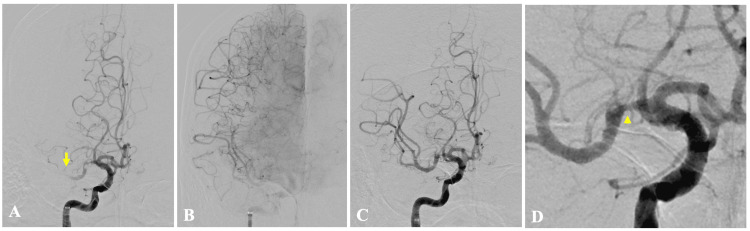
First mechanical thrombectomy Preoperative right internal carotid angiography shows a filling defect in the M1 segment of the middle cerebral artery (A, arrow) and development of retrograde collateral circulation (B). Postoperative angiography demonstrates recanalization (C) and reveals residual M1 stenosis (D, arrowhead)

MT was performed using a combined technique with the SOFIAFLOW Aspiration Catheter (MicroVention, Aliso Viejo, CA, USA) and the Tigertriever revascularization device (Rapid Medical, Yokneam, Israel), achieving complete reperfusion with thrombolysis in cerebral infarction (TICI) grade 3 (Figures 2A-2C). Angiography revealed the development of retrograde collateral circulation, a finding characteristic of ICAD (Figure 2B). MT revealed underlying focal stenosis of the right MCA (Figure 2D), which persisted after recanalization. Given its fixed appearance, the lesion was considered more consistent with ICAD than with an in situ thrombus or vasculitis, directly informing the decision to proceed with rescue angioplasty. A loading dose of aspirin (200 mg) was administered.

Postoperatively, edoxaban 60 mg and prasugrel 3.75 mg were continued, and aspirin 100 mg was added. Laboratory tests on admission showed thrombocytopenia, with a platelet count of 51 × 10^3^/μL (normal range: 140-379 × 10^3^/μL). According to the disseminated intravascular coagulation (DIC) diagnostic criteria proposed by the Japanese Society on Thrombosis and Hemostasis, overt DIC was excluded (Table 1) [7].

**Table 1:** Laboratory test results on admission Laboratory tests on admission excluded disseminated intravascular coagulation

Laboratory tests	Results	Normal range
Aspartate aminotransferase (U/L)	36	10-40
Alanine aminotransferase (U/L)	22	5-45
Creatinine (mg/dL)	1.15	0.65-1.09
Blood urea nitrogen (mg/dL)	39.0	8-20
C-reactive protein (U/L)	2.24	<0.30
Red blood cell count (×10^6^/μL)	395	4.38-5.77
Hemoglobin (g/dL)	11.4	13.6-18.3
White blood cell count (/μL)	11260	3500-9700
Platelet count (×10^3^/μL)	51	140-379
Activated partial thromboplastin time (sec)	34.5	26-38
Prothrombin time-international normalized ratio	4.52	0.90-1.13
Fibrinogen (mg/dL)	179	170-410
Fibrin/fibrinogen degradation products (μg/mL)	5	<5
Antithrombin (%)	92	80-130
Thrombin-antithrombin complex (ng/mL)	15	<4.0
D-dimer (μg/mL)	5.3	<1.0

**Figure 3:**
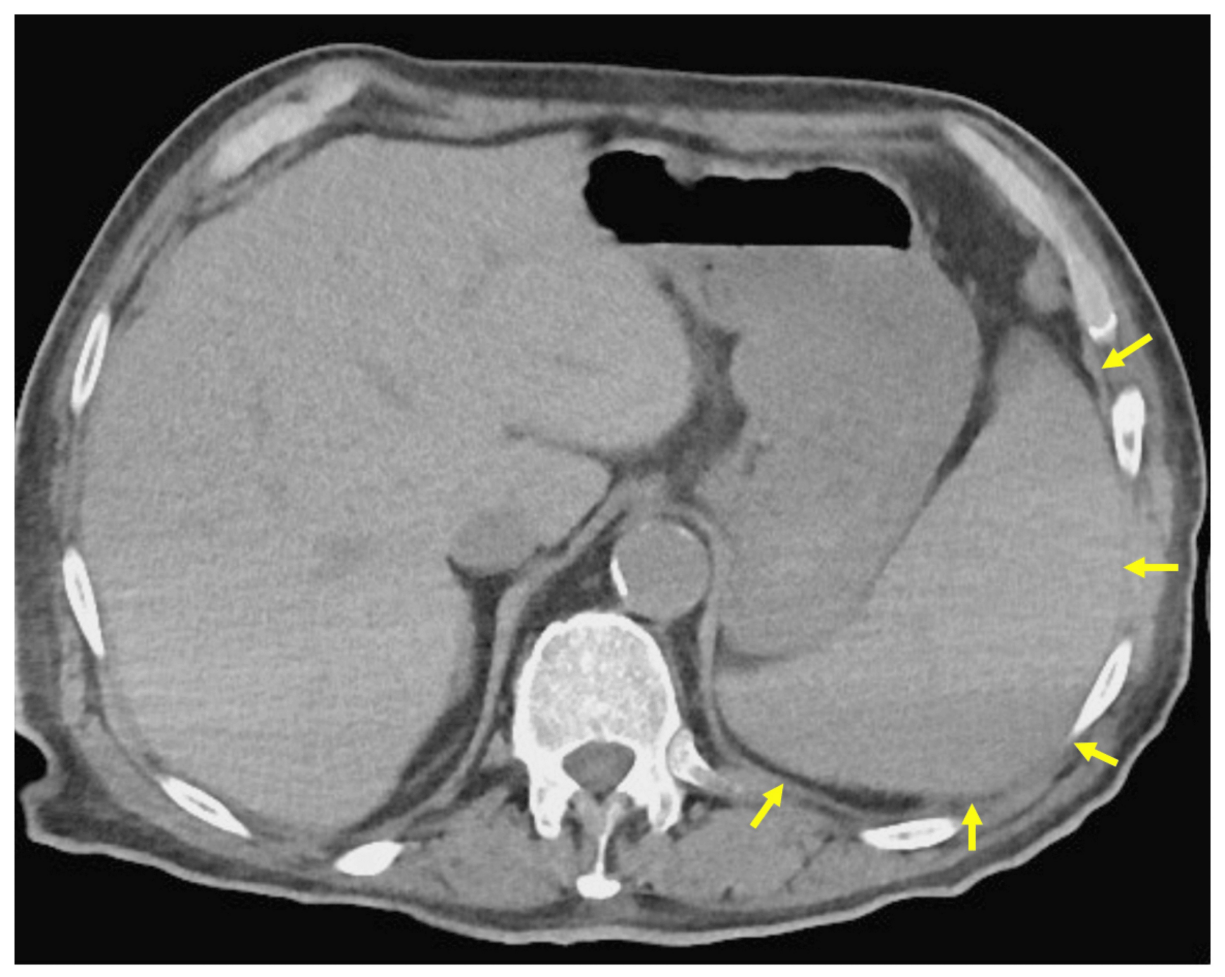
Abdominal computed tomography An axial abdominal computed tomography image reveals splenomegaly (arrow)

A whole-body CT performed after MT demonstrated splenomegaly (Figure 3).

**Figure 4:**
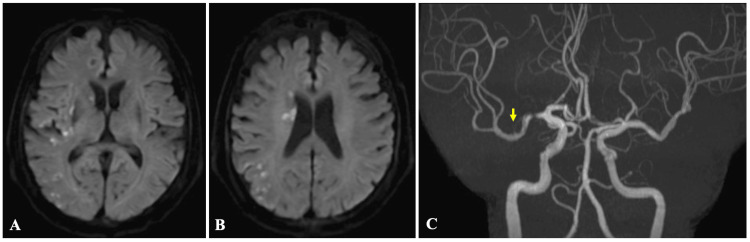
(A and B) Brain magnetic resonance imaging and (C) angiography at day 2 Axial diffusion-weighted images show no new hyperintense lesions and stable findings in the M1 segment of the middle cerebral artery territory (A and B). Magnetic resonance angiography demonstrates preserved patency in the M1 segment of the right middle cerebral artery, indicating maintained recanalization (C, arrow)

On day 2, a follow-up MRI showed stable infarction without progression and patent revascularization (Figure 4).

**Figure 5:**
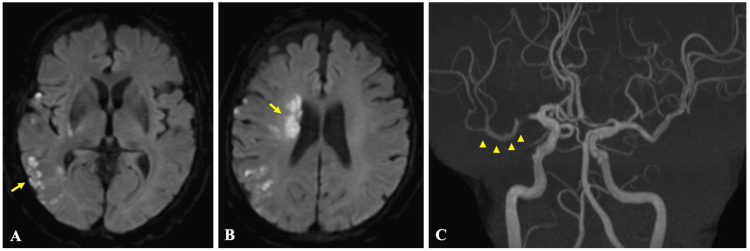
(A and B) Brain magnetic resonance imaging and (C) angiography at day 13 Axial diffusion-weighted images show new hyperintense lesions involving a portion of the right middle cerebral artery territory (A and B, arrow). Magnetic resonance angiography demonstrates that the M1 segment is nearly occluded with decreased distal flow signal beyond the stenotic segment (C, arrowhead)

On day 4, pancytopenia had progressed rapidly, and the platelet count had decreased to 23 × 10^3^/μL. On day 5, a hematologist evaluated the patient and performed bone marrow aspiration and biopsy, which led to the diagnosis of Philadelphia chromosome-positive ALL. On day 8, prednisolone 60 mg was initiated as part of induction therapy for Philadelphia chromosome-positive ALL. On day 9, worsening anemia due to hematuria led to the temporary discontinuation of dual antiplatelet therapy (DAPT), and edoxaban alone was continued. On day 13, left hemiparesis and dysarthria recurred, and MRI showed an extension of the right cerebral infarction and reocclusion of the M1 segment of the right MCA (Figure 5).

**Figure 6:**
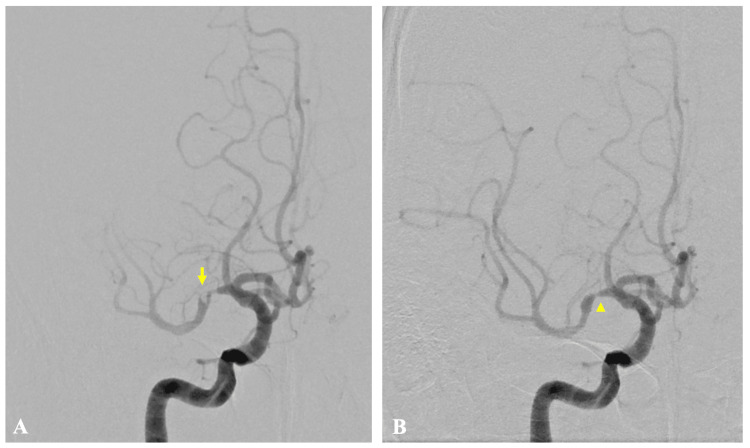
Second mechanical thrombectomy Preoperative right internal carotid angiography shows a recurrent filling defect in the M1 segment of the right middle cerebral artery with delayed distal flow (A, arrow). Postoperative angiography demonstrates recanalization and again reveals residual M1 stenosis (B, arrowhead)

DAPT was resumed before the second MT. A second MT was performed using a combined technique with the AXS Vecta Aspiration Catheter (Stryker Neurovascular, Fremont, CA, USA) and the EmboTrap revascularization device (CERENOVUS, Johnson & Johnson Medical Devices, Irvine, California, USA), achieving reperfusion with TICI grade 3 (Figure 6).

**Figure 7:**
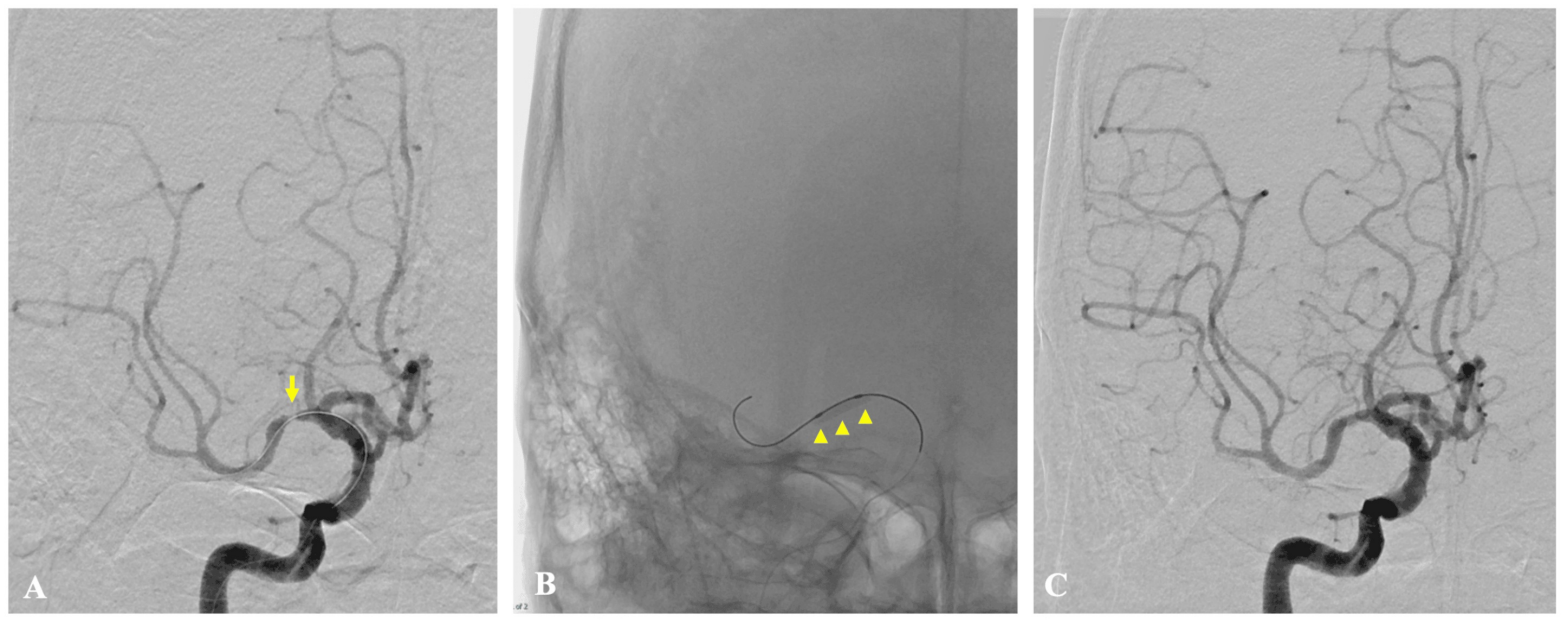
Intracranial percutaneous transluminal angioplasty Preoperative right internal carotid angiography shows M1 segment stenosis of the middle cerebral artery (A, arrow). Balloon angioplasty results in successful recanalization (B, arrowhead), and postoperative angiography confirms adequate vessel dilatation (C)

The retrieved thrombus was white in appearance, suggesting a platelet-rich component. Histopathologic examination demonstrated a fibrin-dominant, fresh thrombus with admixed degenerated neutrophils. PTA was performed using an Rx Genity 2.5-mm balloon catheter (Kaneka Medix, Osaka, Japan). Balloon inflation was initiated and maintained at 4 atm for one minute, despite the nominal pressure of the device being 6 atm. During this time, angiography confirmed dilation of the stenotic segment, resulting in adequate dilation. After a five-minute observation period, repeat angiography demonstrated sustained vessel patency without evidence of residual stenosis or early recoil (Figure 7).

**Figure 8:**
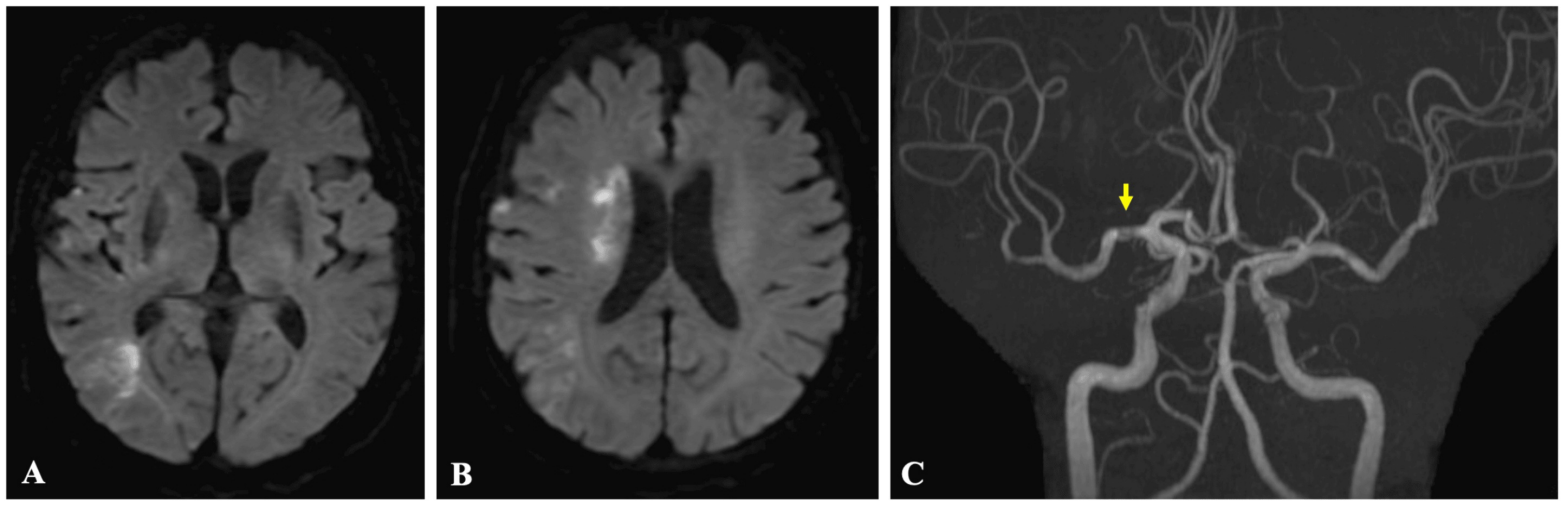
(A and B) Brain magnetic resonance imaging and (C) angiography at discharge Axial diffusion-weighted images show no new ischemic or hemorrhagic lesions and stable findings in the right MCA territory (A and B). Magnetic resonance angiography demonstrates preserved right M1 patency without evidence of dissection or restenosis, indicating maintained recanalization (C, arrow)

No flow-limiting dissection or distal embolization was observed. Although left upper-limb weakness persisted, his neurological condition gradually improved. He experienced no perioperative complications. On day 21, he was transferred to another facility for further induction therapy for ALL with an mRS score of 4 (Figure 8).

## Discussion

In the present case, the patient suffered an LVO despite being treated with prasugrel and the oral anticoagulant edoxaban. Whole-body CT performed as part of a malignancy workup revealed splenomegaly, which subsequently led to the diagnosis of ALL. Based on angiographic findings of retrograde collateral circulation and persistent focal stenosis, the lesion was diagnosed as ICAD. The patient was receiving DAPT for secondary prevention of symptomatic ICAD. However, therapy could not be continued because of severe ALL-related bleeding, which led to recurrent ischemic stroke. Because DAPT was expected to be difficult to continue during ALL treatment due to bleeding tendency, we performed intracranial PTA in the acute phase. This strategy achieved a favorable outcome. This case provides two important clinical implications. (1) A diagnostic implication is that, when LVO occurs despite multiple antithrombotic therapies, malignancy-associated coagulopathy should be considered, and whole-body evaluation should be performed. (2) A therapeutic implication is that, in cases of symptomatic ICAD with bleeding tendency that makes continuation of antithrombotic therapy unfeasible, acute-phase intracranial PTA may be a viable treatment option.

First, when LVO occurs despite multiple antithrombotic therapies, malignancy-associated coagulopathy should be considered, and a whole-body CT should be performed to evaluate for underlying neoplastic disease. Recent reports have shown that approximately 6.8% of patients undergoing endovascular thrombectomy for acute ischemic stroke have underlying malignancy, indicating that cancer is an important cause of ischemic stroke [8]. Malignancy induces tumor cell-induced platelet aggregation, which directly contributes to a prothrombotic state [9]. Thrombotic complications have also been reported in ALL [10]. In this case, the occurrence of LVO while the patient was taking prasugrel and edoxaban was difficult to explain by routine atherosclerotic mechanisms, which led to a whole body evaluation as the decisive trigger. These findings suggest that both malignancy-associated hypercoagulability and local ICAD likely acted synergistically in this patient. In clinical practice, when ischemic stroke occurs despite multiple antithrombotic therapies, one should consider not only drug resistance but also the possibility of coagulation abnormalities related to underlying disease, and early malignancy screening, including whole-body CT, is advisable.

Second, when newly diagnosed ALL is accompanied by a significant bleeding tendency that makes continuation of antithrombotic therapy difficult, acute-phase intracranial PTA may be a viable alternative for preventing recurrence of symptomatic ICAD. In ALL, thrombocytopenia resulting from bone marrow suppression and enhanced fibrinolysis associated with disseminated intravascular coagulation can make continuation of antiplatelet or anticoagulant therapy difficult [11]. In this case, antithrombotic therapy had to be discontinued because of hematuria-related anemia, after which symptomatic ICAD recurred. In Philadelphia chromosome-positive ALL, BCR-ABL1 inhibitors have proven effective, and the introduction of dasatinib has improved long-term outcomes [12]. However, gastrointestinal bleeding has been reported in approximately 10% of patients treated with dasatinib [13]. Therefore, interruption of antithrombotic therapy may be required. When the continuation of antithrombotic therapy is uncertain, establishing a strategy to prevent the recurrence of symptomatic ICAD becomes crucial. MT remains the first-line treatment for acute LVO; however, underlying ICAD is a major cause of procedural failure and early reocclusion. MT failed in 11.9% of cases, and 70% of these failures were attributable to ICAD [14]. ICAD-LVO was associated with a significantly higher reocclusion rate of 36.9% [15]. The optimal acute-phase management of symptomatic ICAD remains an important clinical issue [2,3]. However, the use of acute-phase submaximal balloon angioplasty has been reported as a possible treatment option [4]. Intra-procedural complications related to PTA occurred in 17.4% of cases, including vasospasm, arterial dissection, pseudoaneurysm, arterial occlusion, arterial perforation, arterial rupture, hemorrhage, and thrombosis, with arterial dissection being the most frequent. In addition, restenosis within one year was observed in 15.7% of cases(4). Although these complications are possible, when adequate medical therapy cannot be administered, especially in cases like this where interruption of DAPT is highly anticipated during induction therapy for ALL, selecting acute-phase intracranial PTA can be considered clinically reasonable. In this case, acute-phase intracranial PTA was performed without perioperative complications, and the patient’s neurological symptoms improved.

To our knowledge, there are few reports addressing secondary prevention strategies for ICAD-related LVO in patients with a high bleeding risk, such as those with ALL, in whom antithrombotic therapy is not feasible. Several limitations should be acknowledged. First, this report describes a single case, limiting generalizability. Second, evidence regarding secondary prevention strategies for ICAD-related LVO in patients with malignancy-associated bleeding risk remains extremely limited. Despite these limitations, the present case suggests that revascularization strategies may have a potential role in selected patients with cancer-associated arterial thrombosis when standard antithrombotic therapy cannot be safely continued.

## Conclusions

This case suggests that when LVO occurs during multiple antithrombotic therapies, malignancy-associated coagulopathy should be suspected early, and whole-body imaging should be promptly performed. It also indicates that acute-phase intracranial PTA may be a therapeutic option for symptomatic ICAD when continuation of antithrombotic therapy is difficult because of bleeding tendency. This case uniquely illustrates the potential role of acute-phase intracranial PTA as a rescue strategy for recurrent LVO when antithrombotic therapy is contraindicated, highlighting its novelty and possible clinical value in highly complex scenarios. These points provide important implications for the management of arterial thrombosis in patients with underlying malignancy. The main limitation of this report is that it describes a single case; therefore, the relevance of malignancy-related coagulopathy and the applicability of acute-phase PTA cannot be generalized. Further accumulation of cases is needed to evaluate the safety and effectiveness of acute-phase PTA for symptomatic ICAD in patients with malignancy-associated arterial thrombosis, especially when adequate antithrombotic therapy cannot be administered.
